# Archaeometabolomics characterizes phenotypic differences in human cortical bone at a molecular level relating to tobacco use

**DOI:** 10.1126/sciadv.adn9317

**Published:** 2024-10-04

**Authors:** Diego Badillo-Sanchez, Anna M. Davies-Barrett, Maria Serrano Ruber, Donald J. L. Jones, Sarah A. Inskip

**Affiliations:** ^1^ School of Archaeology and Ancient History, University of Leicester, Leicester LE1 7RH, UK.; ^2^ Leicester Cancer Research Centre, RKCSB, University of Leicester, Leicester LE1 7RH, UK.; ^3^ The Leicester van Geest MultiOmics Facility, University of Leicester, Leicester LE1 7RH, UK.

## Abstract

Tobacco consumption affects human health, but no studies have investigated its effect on the bone metabolome, or if any changes are traceable after long postmortem intervals. Human osteoarchaeological remains preserve small molecules, making them valuable for studies that aim to examine past conditions. We test if there are molecular differences in the metabolome of cortical bone between archaeological individuals who used tobacco and those who did not, and if these differences are distinct enough to assign tobacco use status to individuals with unknown tobacco use. Cortical bone of 323 known and unknown tobacco users was studied by an untargeted metabolomics assay using a liquid chromatography–high-resolution mass spectrometry platform. We identified 45 discriminating molecular features that differed between tobacco consumers (15 up-regulated features) and nonconsumers (17 up-regulated features). Tobacco consumption leaves a metabolic record in human bone distinctive enough to identify its use in individuals of unknown tobacco consumption. Future work will validate molecular features relating to tobacco consumption.

## Teaser

Archaeometabolomics identifies differences in cortical bone metabolome that correlate with tobacco use.

## INTRODUCTION

Tobacco, a natural plant (*1*) used by humans for millennia (*2*–*4*), has been globally available since the late 16th century (*5*, *6*). Various tobacco species, primarily *Nicotiana rustica*, *Nicotiana tabacum*, and *Nicotiana quadrivalvis*, are and have been consumed through smoking, chewing, snussing, or snuffing in varying quantities across the globe. This consumption introduces a wide range of molecules into the body’s general circulation (*1*, *7*).

Historical records from the 16th to 19th centuries commented on negative and, in some cases, perceived positive effects of tobacco consumption on the body (*8*, *9*). Modern clinical studies have conclusively shown that tobacco consumption negatively affects human health, affecting various organs and tissues. This leads to conditions such as lung, bladder, and throat cancers, increased risk of stroke, and coronary artery disease (*8*, *10*–*14*). Tobacco consumption is also a key risk factor for lung diseases such as chronic obstructive pulmonary disease and emphysema (*7*, *15*). In addition, associations between tobacco smoking and musculoskeletal issues, such as low bone mineral density, increased fracture risk, and periodontitis, have been identified (*16*–*18*).

The health effects of tobacco consumption in past populations are of great relevance in the humanities and health sciences (*5*, *8*, *19*, *20*), yet have received scant attention due to the inability to link tobacco use and disease. As in modern clinical examples, studies on past population health and the risks of tobacco consumption require controlling confounding factors to avoid bias or confusion in conclusions (*11*). Historical records lack individual level data needed to answer questions about the impact of tobacco on past health. However, archaeological human skeletal remains (HSR) and their archaeological metadata have the potential to provide direct evidence that can be used to study past pathological and health conditions (*21*), including diseases associated with tobacco use (*22*).

Tobacco consumption in HSR can be identified visually through osteoarchaeological observation (*23*); a past individual’s’ dentition may show evidence of distinctive wear from tobacco clay pipes (pipe notches) or staining on the inner (lingual) surfaces of the teeth (*23*). This evidence can be used to categorize individuals as detected tobacco consumers (DTCs) or nondetected tobacco consumers (NTCs). However, many archaeological individuals have poorly preserved dental remains or lose their teeth before death, categorizing them as undetermined tobacco consumers (UTCs). It is important to also note that osteoarchaeological observations are specific to certain types of tobacco consumption and do not reflect tobacco use through methods like snuffing or enemas (*24*). In addition, infrequent tobacco use or exposure to secondhand smoke cannot be detected through this visual technique.

Previous research by our group has demonstrated that untargeted archaeometabolomics applied to HSR, using a liquid chromatography–high-resolution mass spectrometry (LC-HRMS) platform, can be used to investigate metabolomic differences in past populations (*22*). In this investigation, we conduct a comparative biomolecular study using DTC and NTC individuals, including control individuals dating before the introduction of tobacco, to evaluate the less polar/apolar metabolic profile in cortical bone and its potential differences due to the effect of tobacco consumption. Subsequently, we create a statistical model using a receiver operating characteristic (ROC) curve to classify UTC individuals as DTC or NTC. Using 323 individuals from two British collections, our biomolecular strategy shows that there are significant differences in the cortical bone metabolome that relate to tobacco consumption status, potentially inferring metabolic changes in human cortical bone, and that these changes are preserved in HSR over time. Furthermore, this approach also allowed us to estimate tobacco use status in HSR with poorly preserved dentitions or those who were consuming in invisible ways. This effectively will allow direct analysis of tobacco use in the past, which can be used to evaluate its relationship with disease. This highly innovative research is the first metabolomic study of tobacco use and human cortical bone in any field, the latter of which is rarely studied in metabolomics due to ethical limitations of sampling living individuals. Last, this study shows that we are able to directly investigate past health parameters from archaeological human cortical bone through metabolomics, offering opportunities to explore past or modern health conditions.

## RESULTS

### Osteoarchaeological classification of HSR

The osteoarchaeological component of the research required the analysis of 323 individuals (Supplementary Methods and data S1). This consisted of 177 adult individuals from the 18th- to 19th-century urban cemetery site of St James’s Garden Burial Ground (SJ) in Euston, London, and 146 individuals from a rural church cemetery site located in Barton-upon-Humber, North Lincolnshire (BH). Individuals from BH were divided into two groups: 45 individuals dating to before the introduction of tobacco to Europe (1150 to 1500 CE) (BH1) and 101 dating after the introduction of tobacco (1500 to 1855 CE) (BH2). Group BH1 posed as a “validation” group to test the model as no individuals should be classified as positive for tobacco consumption because they predate it in Europe. Macroscopic analysis revealed unique postmortem alterations in each archaeological individual, including variation in the total number of bones, extent of bone damage and postmortem fracturing, and alterations to bone composition affecting coloration. However, periosteal (outer layer) bone coloration remained relatively consistent at each location (fig. S2). Specifically, HSR from SJ generally exhibited a blackish coloration, likely due to the presence of soils with high humic acids (*25*, *26*). In contrast, HSR from BH typically displayed a whitish coloration, characteristic of burials in dry, sandy soils (*27*). After conducting osteoarchaeological analysis of all HSR used here (fig. S3), 90 individuals showed macroscopic evidence of tobacco use (DTC) in the dentition (figs. S4 and S5), 68 showed no evidence of tobacco consumption (NTC) (fig. S6), and 153 could not be classified due to poor dental preservation or antemortem tooth loss (UTC) (fig. S7).

### LC-HRMS untargeted metabolomic assay for tobacco detection in HSR

During microsampling of the HSR, taphonomic processes were visible as coloration changes at a periosteal level, but these effects were not discernible in the internal cortical bone; all samples exhibited a similar cream-white tone (fig. S8). However, variation in the texture of the resultant samples between each archaeological location (fig. S9) indicated different degrees of taphonomic alteration in the collagen structure for both sites.

**Fig. 1.**
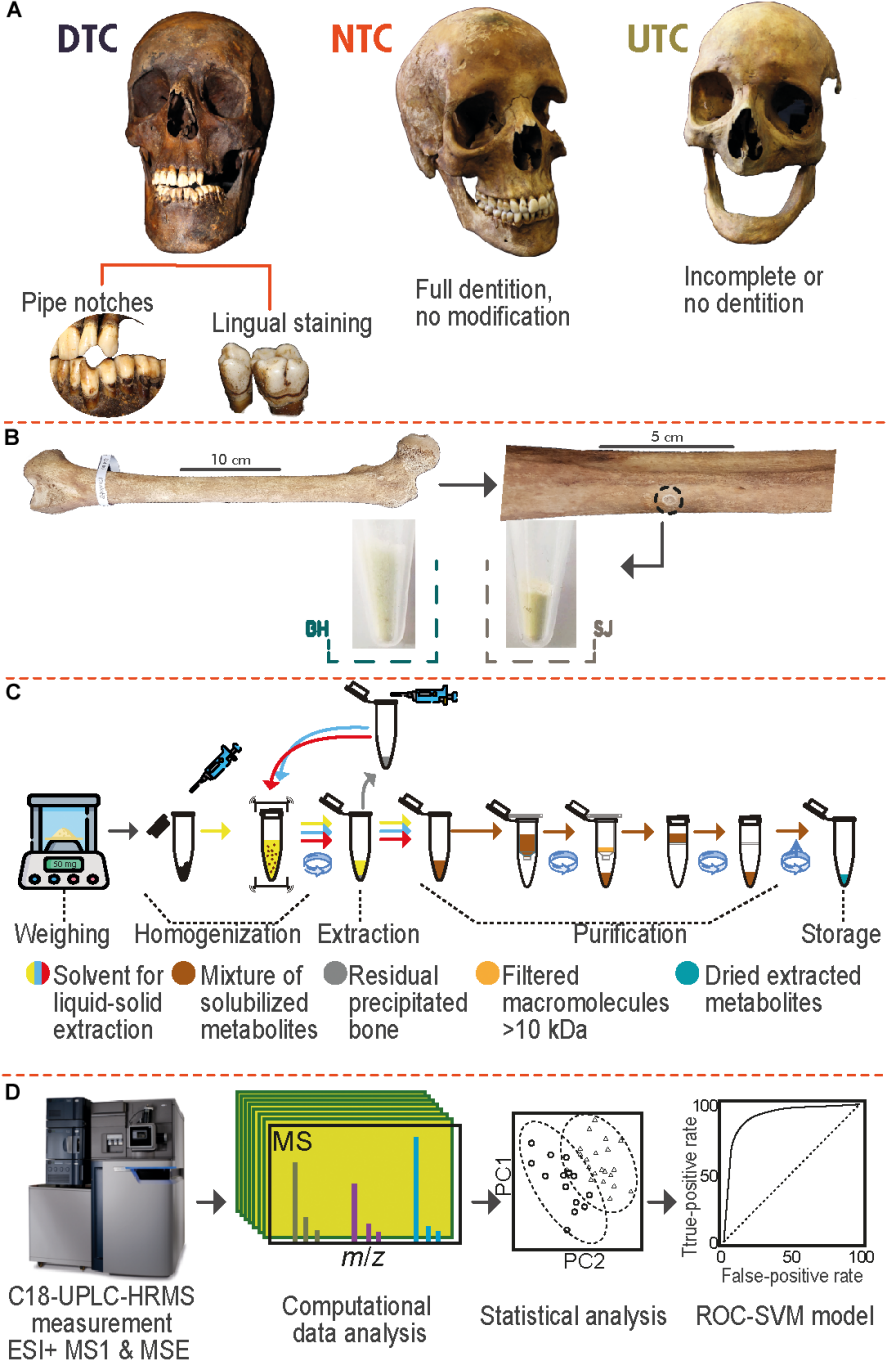
Experimental design followed for the untargeted metabolomic assay. (**A**) Osteoarchaeological inspection and classification of the British HSR; DTC, detected tobacco consumer; NTC, nondetected tobacco consumer; UTC, undetermined tobacco consumer. (**B**) Microsampling detail to obtain cortical bone from femora, example of a resultant sample from Barton upon Humber (BH) and St James’s Gardens Burial Ground, London (SJ). (**C**) Detail of the liquid-solid microextraction process. (**D**) Analytical, postanalytical, and biostatistics steps for untargeted metabolomics profiling.

Using an untargeted archaeometabolomic approach (Fig. 1 and Supplementary Methods), the analysis of DTC, NTC, UTC, and quality assurance/quality control (QA/QC) samples resulted in a total of 3083 molecular features after data preprocessing. The raw metabolomics data matrix initially contained numerous false-positive features, including features with high variance among individuals (>40%), features with a high prevalence of missing values (>80%), or features related to the metabolomics process (extraction, MS measuring, or preprocessing). QA/QC samples indicated that MS data were affected by normal decay and intra- and inter-batch effects typical of a time-of-flight (TOF) analytical platform (figs. S11 and S12). After QA/QC and data processing (Supplementary Methods, figs. S13 and S14), a total of 2958 features were filtered out, leaving 125 features attributable to biological information extracted from the HSR cortical bone. QC data confirmed that the resultant metabolomic information was not altered after data processing and was directly related to the individuals rather than any analytical or experimental effects (fig. S15).

**Fig. 2.**
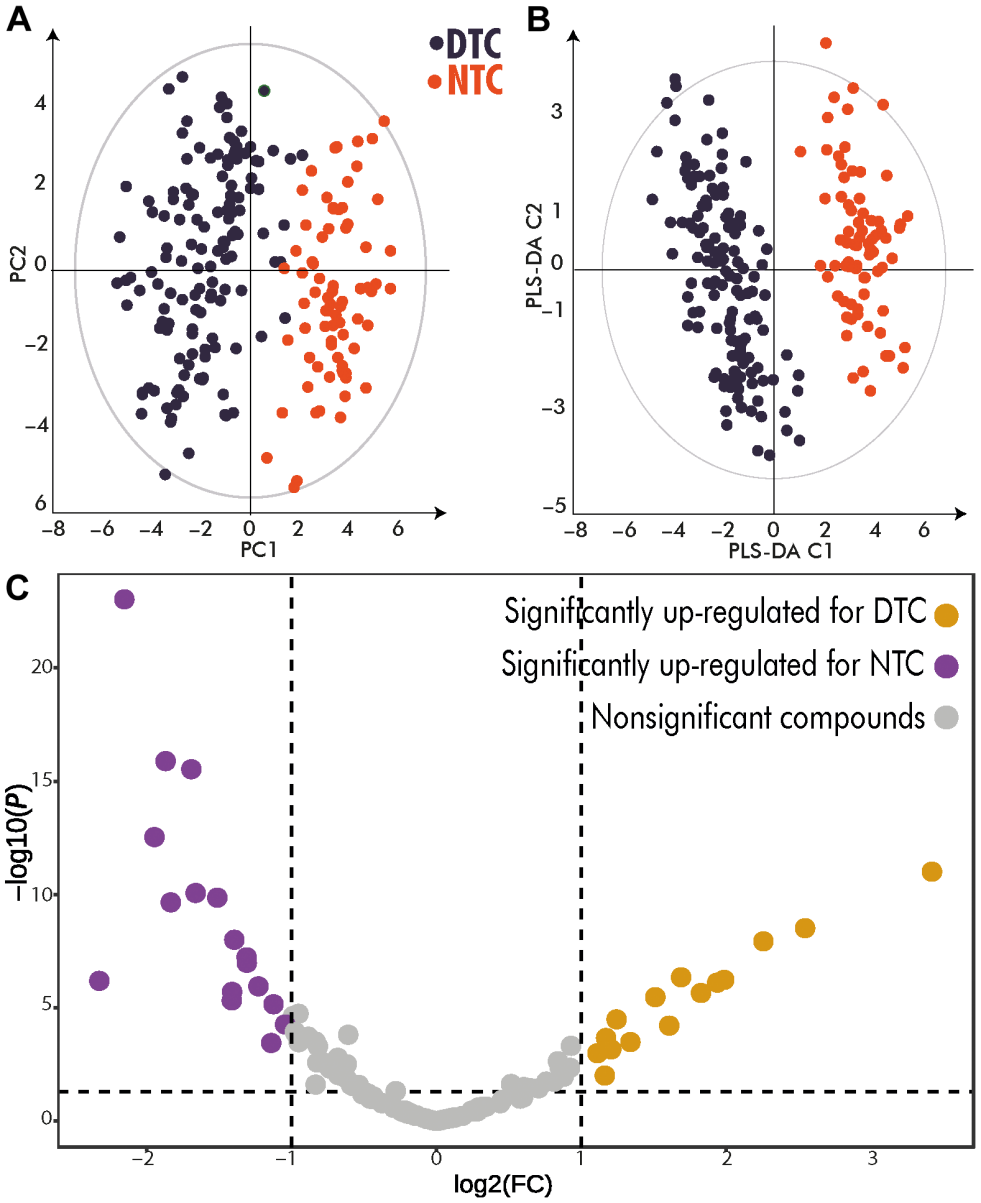
Statistical models applied to the LC-HRMS untargeted metabolomic data from all individuals. (**A**) PCA score plot model *n* = 209, 125 molecular features, PC1, R^2^X: 0.0652; Q^2^: 0.0332; PC2, R^2^X: 0.0406; Q^2^:0.00855. (**B**) PLS-DA model *n* = 209, 125 molecular features, PLS-DA C1, R^2^X: 0.064, R^2^Y: 0.854, Q^2^: 0.826. PLS-DA C2: R^2^X: 0.030, R^2^Y: 0.0542, Q^2^: 0.092. (**C**) Volcano plot for the statistical comparison between DTC and NTC groups.

The unsupervised statistical model using principal components analysis (PCA) to compare the untargeted archaeometabolomic profiles of DTC and NTC samples showed a clear separation between tobacco use conditions along PC1 and PC2 in the respective PCA score plot (Fig. 2A), with a tendency for more of the NTC samples to be toward negative PC2 values. A few DTC samples exhibited behavior more similar to the NTC samples but did not cluster with them. Other possible trends were evaluated by using the available metadata (data S1) and no other clustering based on the variables analyzed showed any contribution or influence on the separation of the DTC and NTC metabolomic profiles (fig. S15).

A supervised statistical model using partial least squares–discriminant analysis (PLS-DA) revealed complete cluster separation between DTC and NTC groups (Fig. 2B). Among the features investigated in the PLS-DA model, 45 molecular features had a variable influence on projection (VIP) greater than 1 (table S1), meaning that they had a statistical correlation (at or below *P* ≤ 0.05) with the analyzed group variable (tobacco use or not), making them suitable as discriminator molecules for distinguishing between tobacco consumers and nonconsumers.

Univariate analysis of the untargeted metabolomic data for DTC and NTC groups identified 17 up-regulated molecules in the NTC group and 15 up-regulated molecules in the DTC group (Fig. 2C and fig. S16).

#### Validation of untargeted metabolomic assay

The validation of the multibatch MS1 untargeted metabolomic assay by a second experiment [using a single batch on an MSe mode (Supplementary Methods)] demonstrated that separation in unsupervised (fig. S17) and supervised (fig. S18) statistical models for the comparison of the metabolomic profiles of DTC and NTC offers equal analytical results on different days. It showed that processing data steps do not alter the molecular information obtained. Replicability of the experiment was confirmed through the presence of chromatographic and spectrometric data with a discrete difference between assays in terms of mass/charge ratio (*m*/*z*) [below 10 parts per million (ppm)], CCS (collision cross section) values (below 5%), and retention time (below 0.2 min). VIP molecules associated with the tobacco consumption were confirmed in the second experiment (MSe) with respect to the MS1 assay.

#### Metabolomic annotation

Metabolomic annotation for VIP features was accomplished using MS and CCS information from MS1 and MSe experiments, allowing for multiple ID hits when compared with available databases (Supplementary Methods and data S3). All were at a confidence of level 3. Further investigation is required to increase the confidence level on the annotation of these unexplored ancient molecules.

### Statistical classification of UTC based on their metabolomic profile

**Fig. 3.**
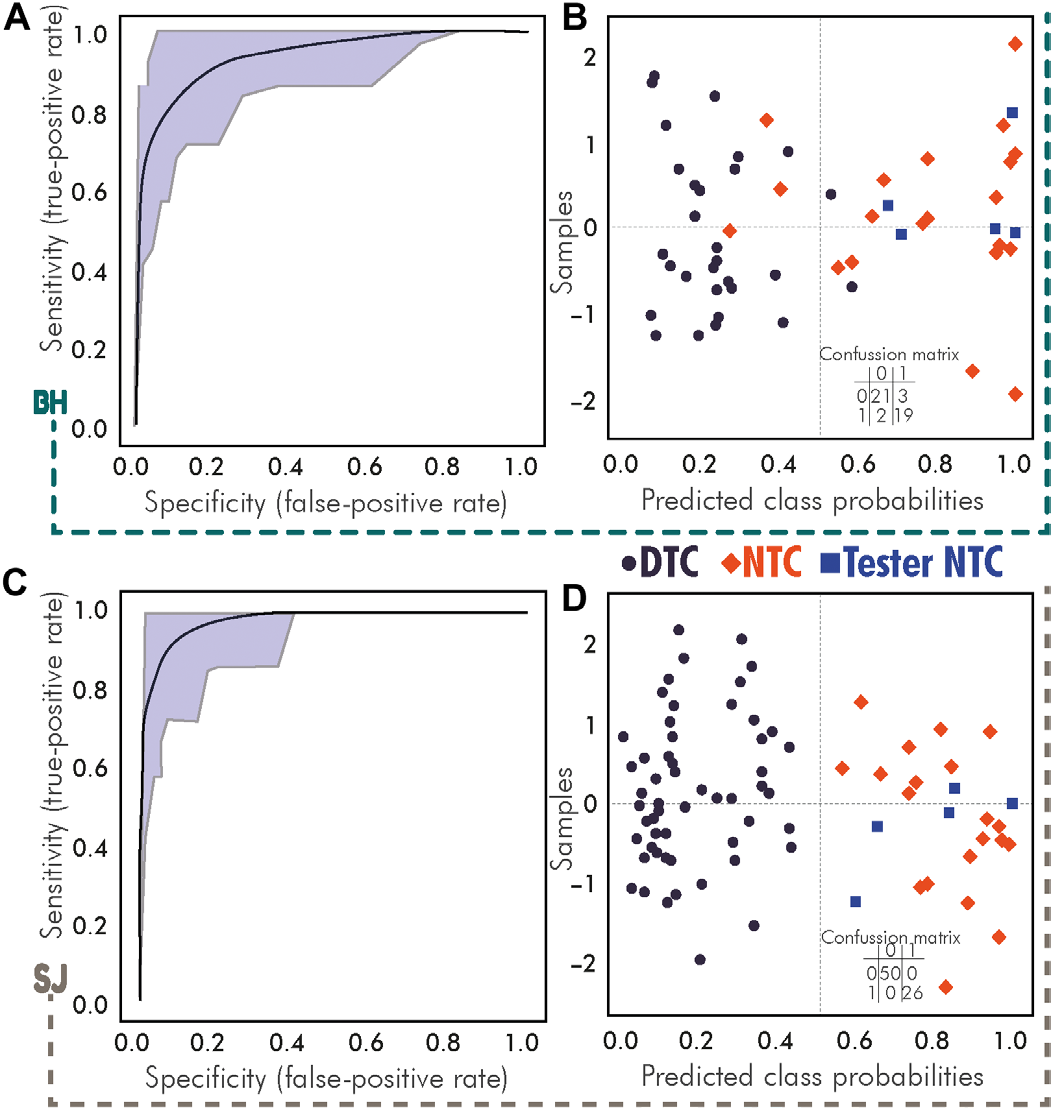
Receiver operating characteristic (ROC) model from molecular VIPs obtained in LC-HRMS untargeted metabolomics assay for less polar/apolar molecules for the assignation of tobacco consumption status to unknown tobacco consumers. (**A**) ROC curve for model containing individuals from the Barton-upon-Humber site. Area under the curve: 0.95; 95% confidence interval: 0.847 to 1; permutation (100×) empirical *P* value: <0.002. (**B**) Predicted class plot after cross-validation of the SVM model for individuals from the Barton-upon-Humber site. (**C**) ROC curve for the model containing individuals from the St James’s Garden Burial Ground, London site. Area under the curve: 0.974; 95% confidence interval: 0.864 to 1; permutation (100×) empirical *P* value: <0.002. (**D**) Predicted class plot after cross-validation of the SVM model for individuals from St James’s Garden Burial Ground. Black circles: Individuals classified as detected. Orange diamonds: Individuals classified as nondetected. Blue squares: Individuals classified as nondetected held out as validation step.

Last, a statistical classification of UTC individuals based on their metabolomic profile involved the use of a linear support vector machine (ROC-SVM) with the 45 VIP features (Supplementary Methods). The results, as shown in Table 1 (table S2 and data S3), indicate that the model applied to BH individuals (rural environment and pretobacco individuals) (Fig. 3A) had a minimal misclassification rate and demonstrated high sensitivity (Fig. 3B). The same model applied to SJ individuals (industrialized environment) (Fig. 3C) displayed no misclassification, high sensitivity, and low specificity (Fig. 3D). An approximately 9% error was found according to the pretobacco validation group samples.

**Table 1.** Assignation of tobacco consumption class based on the SVM model. BH, Barton-upon-Humber; SJ, St James’s Garden Burial Ground, London. BH1, pretobacco period (1150 to 1500 CE); BH2, posttobacco period (1500 to 1855 CE).

Archaeological phase	Total individuals	% Detected tobacco consumers	% Nondetected tobacco consumers
**Barton-upon-Humber**			
**BH1**	23	13	87
**BH2**	61	57.4	42.6
**St James’s Garden Burial Ground, London**			
**SJ**	69	56.3	43.7

## DISCUSSION

This research aimed to assess whether there were significant differences in the metabolome of cortical bone between archaeological individuals who did and did not use tobacco. We have shown that there are distinct differences in the molecular features of cortical bone between tobacco users (DTC) and nonusers (NTC) (including pretobacco controls). Furthermore, these differences are significant enough that they allow us to discriminate between them and allow us to reliably assign tobacco use status to individuals where it is not possible to examine for visible markers of tobacco use. There has been very limited metabolomic research on archeological human cortical bone, and the relationship between tobacco consumption and (human) cortical bone metabolome has been poorly understood. This groundbreaking research shows that archaeometabolomics has a lot to offer in terms of understanding past phenotypes, like tobacco smoking, which can help us better understand health conditions in the past and their relationship to current trends. However, there are a number of important aspects and limitations that need further discussion to move the approach forward, and to make archaeometabolomics a standard approach in the field.

The nature of archaeological material makes it a highly challenging material to work with, which has led us to take a highly cautious approach in data processing. There are often restrictions on the size/weight of samples that can be taken from skeletal material due to their irreplaceability, meaning we are working with concentrations far lower than in, for example, serum. This, in combination with relatively unknown degradation processes and factors relating directly to individual life circumstances, their treatment and location at death, can create a lot of variability in molecular feature data, including many zeros and the low concentration of extracted metabolites that results in low MS detection intensity. Inevitably, this results in a greater attrition rate when applying rigorous cutoffs to manage these uncertainties. This effect is reflected in the workflow algorithm for peak picking, necessitating the removal of a high number of molecules for statistical analysis. Despite this, the features that remained could be linked to a health variable experienced during an individual’s life (in this case tobacco use). A key finding of our study is that although HSR undergo significant postmortem changes, enough usable small molecules are preserved in cortical bone.

The untargeted metabolomics workflow on less polar/apolar molecules extracted from cortical bone at two British archaeological sites allowed us to create metabolomic profiles for the different HSR. Statistical comparisons between individuals who consumed tobacco (DTC) and those who did not (NTC) showed molecular differences in cortical bone. We propose that the metabolomic expression of cells trapped in the interlayers after bone remodeling (*28*, *29*) is influenced by the interaction of tobacco molecules in individuals who consumed tobacco. In addition, the results indicate that in our cohort, when comparing DTC and NTC, there are no distinctions based on age or biological sex, suggesting that bone metabolism in this case is equally affected regardless of sex or age. The untargeted metabolomics discovery approach using an LC-HRMS platform provides a series of molecules that with further work could be used to trace tobacco consumption in archaeological HSR. This approach may also prove valuable in forensic investigations and, once the features are identified, the study of bone-related diseases in clinical trials.

Although candidate molecules were identified (see data S3), we were not able to determine what the features were with certainty as further validation work is needed, especially given the archaeological nature of the material. However, lipids, peptides, and alkaloids, among other classes, were present. The annotation process for ancient molecules yielded a low score compared to available databases, often resulting in a number of hits or none at all. This limitation arises due to the absence of archaeometabolomic studies and specialized metabolomic databases for ancient and even modern bone metabolites. Identifying such molecules becomes challenging without conducting more extensive studies, which necessitate the use of other analytical platforms and larger mass samples. Achieving these requirements in archaeological research is complex due to the irreplaceable nature of the archaeological individuals, which requires ethical justification for their destruction. In addition, costs further restrict the range of experiments that can be conducted, as resources must be allocated to address one research question at a time. Nevertheless, the acquisition of chromatographic and spectrometric data (*m*/*z*, CCS) and a comprehensive metabolomic profile provides valuable insights to address the underlying issues and demonstrates that this is a viable field of research with benefits to archaeology and other allied disciplines.

ROC-SVM models exhibited high accuracy when tested on individuals from the pretobacco medieval period (BH1). Only three individuals from the pretobacco period were classified as DTC. This may have occurred for three reasons: (i) Precisely dating archaeological skeletons is difficult, and it is possible that some skeletons may date a bit later than thought; thus, it is possible that these individuals are among the earliest tobacco consumers. (ii) As we see a possible influence of environmental factors in London, these individuals were exposed to other pollutants that had a similar effect. (iii) Few models based on complex systems are 100% precise. The small number of UTC tested from pretobacco provides the scenario where a very small number of false positives have a disproportionate effect on the overall prediction. Regardless, the models validate the usefulness of the results for further research. Based on the total analysis of the two sample prediction models created, particularly the results of the PLSDA and PCA models, it is evident that the BH model shows better separation than the SJ model. This difference between archaeological locations suggests that, because the investigated molecules probably result from physiological processes, mainly cells trapped in the bone remodeling process, the metabolic processes in these cells differed between the two locations. Individuals from BH lived in a rural environment, while individuals from SJ inhabited an industrialized, highly polluted environment. This may indicate that changes in bone metabolism due to tobacco consumption were more pronounced in the BH population, while people from SJ were also influenced by other factors, possibly including other air pollutants. Such a finding is relevant to modern contexts today, where the impact of environment is increasingly being scrutinized in relation to health. For example, an association between air pollution and increased risk for lower bone mineral density and osteoporosis has been found in modern clinical studies (*30*).

It is worth noting that ROC-SVM modeling required the exclusion of some individuals to improve the models. In most cases, the deleted individuals were those classified as DTC with only lingual staining. In contrast, DTC individuals classified by having pipe notches or a combination of pipe notches and lingual staining were always included in creating the models. This suggests that the method of tobacco consumption, as well as chronicity of use, may have had varying impacts on the observed metabolomic differences, with iterative consumption by pipe use potentially having a greater effect compared to other forms of consumption, such as chewing or the use of cigars. In chewing, for instance, tobacco is not inhaled but enters the body through a different system. Further research would be needed to examine how results might vary between consumption types.

Thanks to the biomolecular results obtained, we were able to use archaeometabolomics to evaluate a human metabolomic condition experienced during life in archaeological individuals. This approach allowed us to classify individuals based on possible tobacco consumption status without the need for well-preserved dentition. Further, this study demonstrates that archaeometabolomics can be used to study past human conditions without being influenced by factors such as the archaeological site, age, biological sex, or analytical steps, such as extraction and injection, when using cortical bone. These results provide osteoarchaeologists with a biomolecular tool that can expand the number of individuals for use in statistical comparisons. Here, combined with osteoarchaeological and paleopathological analysis of diseases in HSR, it is now possible to start to examine how tobacco use in the past affected health and the prevalence of tobacco-related diseases after the introduction of the plant to Europe. Furthermore, this study adds important knowledge to the field as there has been very limited research on the metabolome on human cortical bone, either in archaeology or other biomedical fields, which tend to rely on mice models. As such, the findings are relevant to those in other disciplines, especially those interested in how tobacco use may affect human bone and metabolism.

### Limitations of study and future work

This study was limited by the inherent characteristics of the samples and the limited availability of bone for extraction. Because of the archaeological value and ethical implications of performing destructive analysis on archaeological human remains, it was necessary to obtain the smallest possible sample quantities, which resulted in low concentrations and attrition of data. Going forward, we will gather additional information from the same individuals using HILIC-HRMS to characterize the polar metabolomic profile of the HSR, which may provide valuable additional information without the need to take more material. Future work may consider using more material if it is available and curators are agreeable.

At this present time, we are only able to identify molecular features to level 3. This means that we only have tentative structures for the features responsible for creating the difference between tobacco users and nonusers. Future work is now aiming to validate some of these features so that they can be targeted in future analysis of smoking status. We are currently repeating the experimental process out on a second independent population to identify which features are key in creating the differences between the groups, and these will be taken forward for validation to create a targeted approach.

To move the field of archaeometabolomics forward, work needs to be carried out on archaeological material with known information and provenance (e.g., soil types, date at death, life conditions, etc.) to help populate databases with molecular information that can be used to understand the impact of diagenesis, time since deposition, geology, etc.

## MATERIALS AND METHODS

### Archaeological samples

We performed an osteoarchaeological inspection of 323 adult archaeological individuals (data S1) to define the groups to be used in the untargeted metabolomic assay and subsequent statistical models. We opted to use individuals who were around and over the age of 18 years to limit the number of variables in the bone metabolically. Given the rapid changes in the skeleton during growth, it is likely that there would be differences in the metabolome between children and adults. Two archaeological human skeletal collections from the United Kingdom were used to perform the untargeted metabolomic study (representative image of skeletons in fig. S2). BH and SJ were chosen to evaluate tobacco consumption in British populations. BH consisted of burials from a rural community, which included individuals from the pretobacco medieval period (before the 16th century) and posttobacco period (after the 16th century), and SJ consisted of individuals who lived in the center of industrial urban London (18th to 19th century). Both biological sexes (female and male) with an age range from young adult to late adult (18 to 50+ years) were investigated (fig. S1).

All individuals were subjected to a complete osteoarchaeological analysis. According to the presence or absence of pipe notches and lingual staining (figs. S4 to S6), or incomplete preservation of the dentition (fig. S7), each individual was assigned to one of the following groups: dentition with the presence of pipe notches and/or lingual staining, DTC; dentition without the presence of pipe notches and/or lingual staining, NTC; and individuals with incomplete dentition, UTC. Individuals who were from the pretobacco period were automatically NTC.

### Microsampling of cortical bone

Microsampling was performed following the protocol in Badillo-Sanchez *et al.* (*22*) to obtain a total of 504 bone samples from the different individuals studied (fig. S8 and data S1). Microsampling of cortical bone was performed in a clean fume cabinet as follows: (i) A circular mark to delineate the sampling area of each femoral bone was made 10 mm below (inferior to) the midshaft on the anterior surface using a 6.4-mm-diameter glass drilling bit attached to a rotatory tool operated at 5000 rpm. (ii) The periosteal bone surface of the marked area was microdrilled using a high-speed cutter accessory of 4.8 mm diameter attached to a rotatory tool operated at a speed between 5000 and 10,000 rpm. Drilling continued until the inner cortical bone (~1 to 2 mm in depth) was exposed. Removed bone was collected and properly discarded. (iii) Exposed cortical bone was drilled, as in step ii, until ca. 70 mg of cortical bone was obtained (fig. S9). Care was taken to avoid sampling the endosteal surface or drilling outside of the marked area. Cortical bone samples were collected and stored in an empty labeled 1.5-ml plastic tube. Biological replicates (triplicates) were extracted for a third of the individuals. Microsampling of cortical bone creates a perforation of 6 mm in diameter and variable depth in the skeletal element.

### Filters, vials, and solvents for metabolite extraction and LC-HRMS measurement

All operations were performed by using chemical reagents of optima LC-MS grade. Solvents and formic acid were obtained from Sigma-Aldrich (Poole, Dorset, UK) or Thermo Fisher Scientific (Loughborough, Leicestershire, UK). Total recovery LC-MS vials were purchased from Waters (Elstree, Hertfordshire, UK). Corning Costar Spin-X centrifuge tube filters with cellulose acetate membranes of 0.22 μm (Corning Inc., USA) were purchased from Merck.

### Metabolite extraction of small molecules from cortical bone samples

All samples were randomized and 40 mg of each sample was subjected to a metabolite extraction following the protocol from Badillo-Sanchez *et al.* (*22*) for less polar/apolar features. Metabolite extraction from cortical bone samples were performed in a multistep liquid–solid process as follows: (i) 40-mg aliquots of sampled bone were placed in a new 1.5-ml plastic tube with an O-ring cap and six ceramic beads (ZrO_2_) of 3 mm diameter. Cold methanol (200 μl) was then added. (ii) Tubes were vortexed for 10 s and then placed in a BeadBlaster 24 Microtube homogenizer (Benchmark Scientific Inc., USA) for a sequence of 20 s of homogenization plus 25 s of pause for six cycles. (iii) Tubes were centrifuged for 5 min at 9500 relative centrifugal force (RCF). (iv) The supernatant was pipetted out and stored separately. (v) To the initial tube with the precipitate and the ceramic beads, 200 μl of ethanol was added and steps ii to iv were repeated. The supernatants collected were mixed. (vi) To the initial tube with the precipitate and the ceramic beads, 200 μl of water was added and steps ii to iv were repeated. Resultant supernatants were mixed. (vii) Extracts were filtered through a Microcon 10-kDa centrifugal filter unit—prerinsed with water—in a centrifuge for 20 min at 14,000 RCF. (viii) To the initial tube with the precipitate and the ceramic beads, 200 μl of water was added and steps ii and iii were repeated. The supernatant was decanted and added to the Microcon 10-kDa centrifugal filter and step viii was repeated. (ix) Filtered samples were centrifuged and filtered through a 0.22-μm membrane (Corning Costar Spin-X). (x) Resultant filtrated extracts were placed in a speed vacuum for 2 hours and then frozen with liquid nitrogen to be freeze-dried overnight. Dried extracts were stored at −80°C. Extraction blank samples were prepared to control the process and guarantee the data quality.

### Sample reconstitution for metabolomic LC-HRMS measurements

To reconstitute the metabolite extracts, a volume of 40 μl of a mixture of methanol-water 10:90 was used. Liquid solutions were vortexed for 30 s and centrifuged at 20,000 RCF for 1 min at 4°C to be transferred in a total recovery glass vial before LC-MS measurement.

### Quality assurance

QA and QC processes, as suggested elsewhere, were performed in our untargeted metabolomic process (*24*–*28*) to ensure the quality of the metabolomic study. QA procedures were carried out during all workflow steps to reduce the unwanted variation regarding the preanalytical, analytical, and postanalytical phases of metabolomics.

A set of reference samples, which allow us to evaluate the data obtained and control the performance of the metabolomic process, were prepared. First, three pooled_class_QC were prepared by mixing 5 μl of each sample per group (QC_DTC, QC_NTC, and QC_UTC). Second, a pooled QC sample was prepared by mixing equal volumes of QC_DTC and QC_NTC. Similarly, reference material standard mixes were used to control the column’s performance through the different assays (QC_Col: Reversed-Phase QC Reference Material, Waters). Randomized injections of samples bracketed by pooled QC samples (fig. S10) were included in the analytical assay during the LC-HRMS measurements.

### Metabolomic assays

To obtain the greatest amount of molecular information possible from the osteoarchaeological samples, two different assays were performed to obtain the MS information for the metabolomic profile of the samples for the less polar/apolar extracts. Metabolomic assays were performed in batches composed of biological and QA samples (instrumental blanks, extraction blanks, Class_QC, Column QC, and pooled QC) in a sequence of bracketing every 9 to 10 biological samples by pooled QCs (fig. S10). MS1 measurements were performed on six batches including all samples and replicates. MSe measurements were performed in the extracts from DTC and NTC groups (without replicates) on a single batch.

### Untargeted metabolic fingerprinting by high-flow UPLC-IM-TOF-HRMS

Untargeted analyses were performed on a high-flow Acquity UPLC system coupled to a Waters Synapt G2 HDMS system (Waters Corporation, Manchester) composed of an electrospray ionization source operated in positive mode, an ion mobility (IM) cell, and a TOF detector. MS data were collected using the mobility MS1 or mobility MSe function in profile mode (full scan) over the *m*/*z* 50 to 1500 range. Capillary voltage was set at 3.00 kV, sampling cone 30 V, source temperature 120°C, desolvation temperature 600°C, and desolvation gas 1000 liters/hour. IM and TOF cells were calibrated in advance and the lock spray of leucine enkephalin was used and infused at 10 μl/min. Chromatographic separations were carried out by using a reversed-phase (RP) C18 column [Waters Acquity UPLC BEH C18 column (2.1 × 100 mm, 1.7 μm)] for less polar/apolar metabolites. Samples were maintained at 4°C in the auto sampler and aliquots of 5 μl injected per run. The flow rate was set at 0.4 ml/min, and the column temperature was maintained at 40 ± 2°C. Solvents used for separation consisted of 0.1% formic acid in water as mobile phase A, and 0.1% formic acid in acetonitrile as mobile phase B. The RP gradient elution program started running at 2% B, held for 1 min, and then mobile phase B was linearly increased to 20% to minute 5, 25% in minute 6, 75% in minute 7, and 98% in minute 7.5, then held at 98% of B until minute 8, then reverting to initial conditions of 98% of A in minute 9, and equilibrated the column until minute 10.

### Data treatment

An initial preprocessing treatment—run alignment, ion detection, peak picking, isotope and adduct deconvolution, ion drift measurement, etc.—was performed on the raw data from the different LC-HRMS measurements to obtain the compound metabolomic matrices for the different experiments.

Preprocessing treatment was performed using Progenesis-QI software (Nonlinear Dynamics). Workflows were processed including, on each experiment, the biological samples and QA samples (i.e., QC, extraction blanks, and injection blanks) from the six batches. Resultant data matrices containing the different samples and the total list of features detected for each experiment were split by batch, and each matrix was inspected manually by using Microsoft Excel to filter out nonbiological information (noise, instrumental signals, features from the extraction process, etc.). From our QC and QA tests, we were able to use features identified in extraction blanks, injection blanks, and QC runs to identify and remove unrelated features from the data produced by the actual samples (e.g., plastics, reagents, etc.). We also removed synthetic modern features by using the modern databases. In short, the average value was calculated for each feature in the data matrix for the pooled QC, instrumental blank, and extraction blank samples, as well as the relative standard deviation (RSD) for the QC samples. Features with an injection or extraction blank contribution greater than 5% with respect to the average QC sample were removed. Features with a QC RSD value greater than 40% were removed. Matrices filtered were combined and only features present on all batches were preserved.

Before any further statistical analysis, data transformation was applied; K-nearest neighbors based on similar features was used to replace missing values; and mean sample normalization, log10 data transformation, and Pareto data scaling were applied to the different sets of data.

### Batch correction

To improve the quality of the data and allow the comparison of the different investigated samples affected by the normal MS decay and the intra/inter batch effect, the MetaboAnalyst R package was used with the function of “PerformBatchCorrection.” EigenMS algorithm was applied to the filtered matrices with the inclusion of batch number and injection order for the DTC and NTC samples and pooled QCs (*31*–*33*).

### Statistical analysis

To establish the overall differences in the metabolic profiles between the groups studied, multivariate statistical analysis, including unsupervised PCA and supervised PLS-DA, was performed. MetaboAnalyst 5.0 (https://metaboanalyst.ca/) was used for the statistical analysis of all the experiments (*34*, *35*), as well as SIMCA version 14.0 (Umetrics) software for confirming the results for the untargeted metabolomic analysis. The quality of the established statistical models was evaluated using the *R*^2^*X*, *R*^2^*Y*, and *Q*^2^ parameters. A permutation test was performed in 100 cycles to evaluate the possible overfitting in the PLS-DA models. PCA models were performed, including QC samples, to evaluate the stability of the analytical system and the data quality, using as indicator the clustering of QC samples in successive injections during the sequences. To visualize important features up-regulated or down-regulated when two selected groups of interest were studied, univariate statistical tests were performed, and volcano plots were drawn by transferring the fold change (FC) value of each substance peak to log2 (FC) and transferring the *P* value (*P* = 0.05) of Student’s *t* test to –log10(*P* value).

### Tobacco consumption class prediction

By using the biomarker analysis tool of MetaboAnalyst 5.0 (*34*, *35*), a ROC analysis binary model was made to assign a classification of tobacco consumption to the UTC HSR. For that, an initial multivariate ROC curve–based exploratory analysis was performed by using the total list of molecular VIPs obtained in the PLS-DA model as variables (table S1) and all individuals from the DTC and NTC groups as observations. The model was followed by using a linear SVM classification method and an SVM built-in feature ranking method. Models were evaluated by their ROC curves and the predicted class probabilities (average of the cross-validation) for each sample using the best classifier based on their area under the curve and its confusion matrix. The binary model was improved by an iterative process removing those samples that presented a misclassification after the Monte Carlo cross-validation, which used balanced subsampling of two-thirds of the samples to evaluate the feature importance, which is then validated on the one-third of the samples that were left out. Last, by using the combination of variables and individuals found on the best-fitted model, an ROC curve–based model evaluation algorithm was applied to predict the class for the UTC samples. A subset of five samples per DTC and NTC group were held out for extra validation purposes. Similarly, as it was not possible for individuals from BH phase 1, who correspond to a pretobacco period, to have been affected by tobacco consumption, those individuals were used as a validation set to evaluate the percentage of precision of the model for the classification of UTC individuals.

### Metabolite putative annotation

Putative annotation of the features identified by the high-flow UPLC-IM-TOF-HRMS measurements on C18 experiments was performed using the data identification window of the Progenesis QI software. Data were compared considering all potential matches using the following libraries: (i) METLIN MS/MS library 2017 for Progenesis QI plugin with a 3-ppm precursor and fragment tolerance for H = 0 to 150; C = 0 to 100; N = 0 to 10; O = 0 to 30; F = 0 to 4; Mg, P, S, Cl, Br, I = 0 to 2; (ii) LipidBlast library plugin with a 3-ppm precursor tolerance and 10-ppm fragment tolerance; and (iii) Metabolic Profiling CCS Library search plugin with a 3-ppm precursor tolerance, 10 ppm fragment tolerance, and 10% CCS tolerance.

### Ethics statement

Ethical approval was granted from the Science & Engineering, Arts, Humanities and Law Research Ethics Committee at the University of Leicester (ref: 28448-si159-ss/ah:archaeology&ancienthistory).
