# Supplementary material for: Archaeometabolomics characterizes phenotypic differences in human cortical bone at a molecular level relating to tobacco use
Source: Sci Adv. 2024 Oct 4;10(40):eadn9317. doi: 10.1126/sciadv.adn9317 (PMC11451522; doi:10.1126/sciadv.adn9317)
Supplement: Supplementary file 1 — Figs. S1 to S18 Tables S1 and S2 Legends for data S1 to S4 [file sciadv.adn9317_sm.pdf]

Science Advances
AAAS

Supplementary Materials for

Archaeometabolomics characterizes phenotypic differences in human cortical bone at a molecular level relating to tobacco use

Diego Badillo-Sanchez *et al.*

Corresponding author: Sarah A. Inskip, s.inskip@leicester.ac.uk

Sci. Adv. **10**, eadn9317 (2024)
DOI: 10.1126/sciadv.adn9317

The PDF file includes:

Figs. S1 to S18
Tables S1 and S2
Legends for data S1 to S4

Other Supplementary Material for this manuscript includes the following:

Data S1 to S4

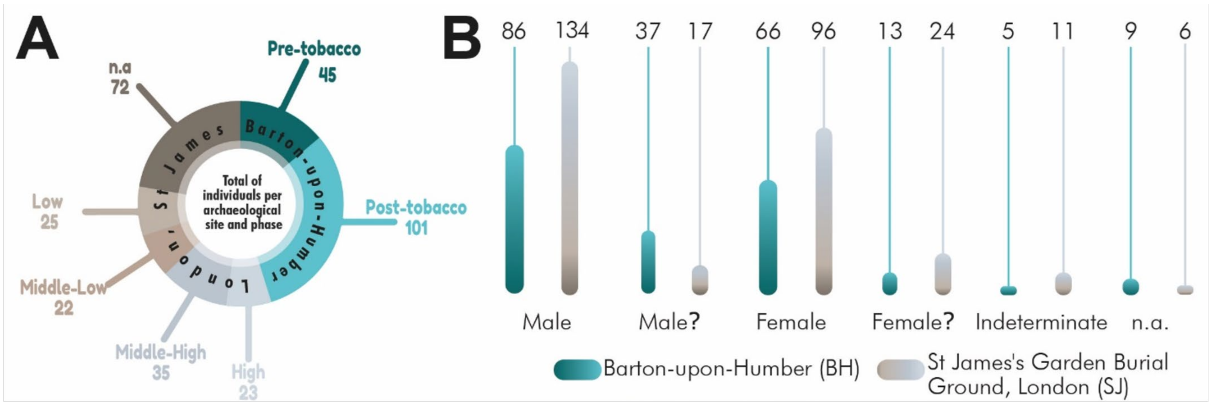

C

Site	Female	Indeterminate	Male	**N.A**	Total
Barton	65	3	71	7	146
St Johns	78	5	90	4	177
Total	143	8	161	11	323

Fig. S1. Samples and percentage of groups according to the archaeological site and biological sex. A) Pie plot for the total number of individuals used for the untargeted metabolomic study. B) Bar plot of frequency of samples according to biological sex. n.a. indicates individuals for whom sex could not be estimated due to poor preservation of the skeleton. C) Table of number of individuals per sex category. N.A = unobservable.

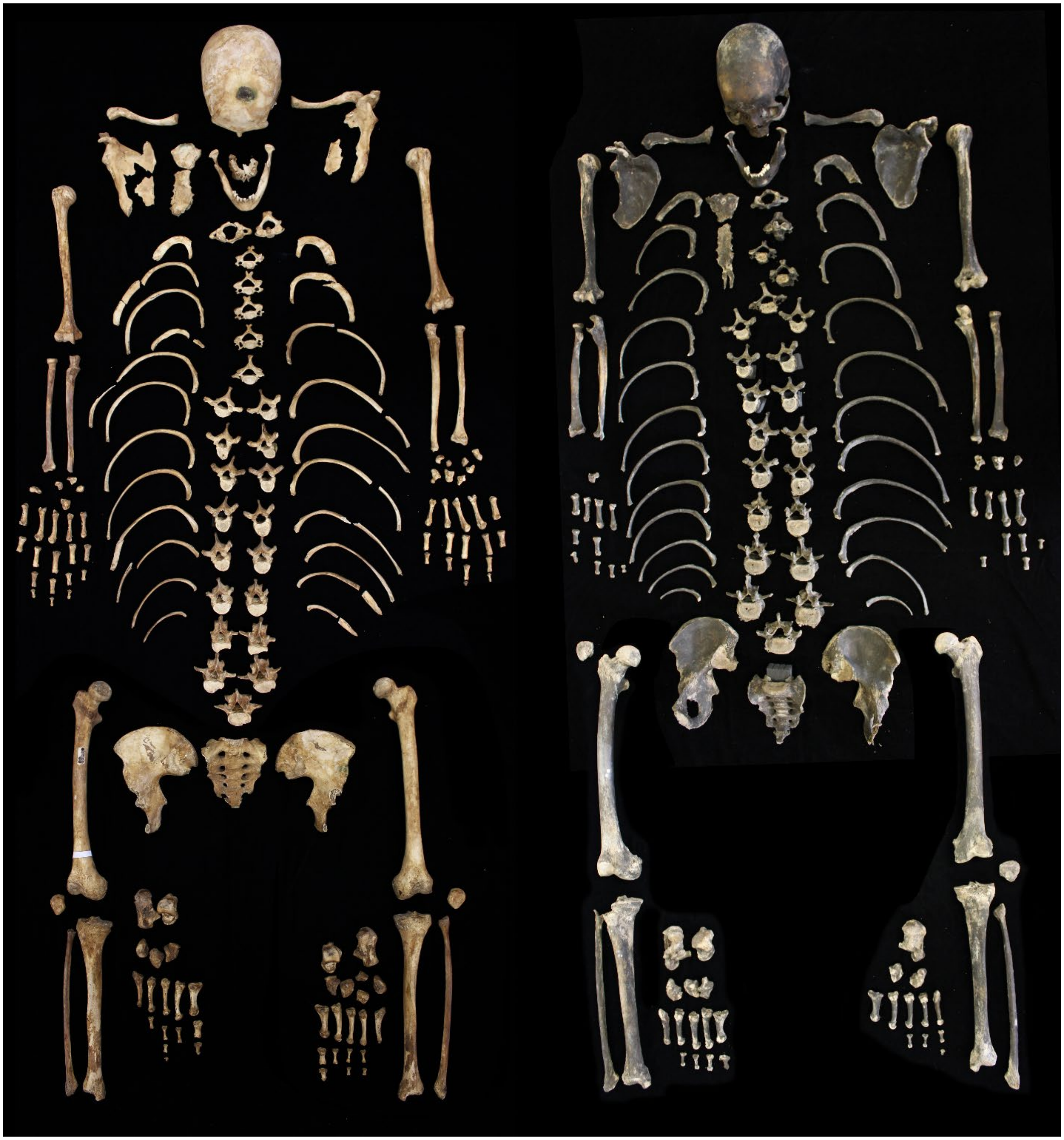

Fig. S2. Examples of complete skeletons from the archaeological sites used for the untargeted metabolomic study.

Left: Example of the average preservation status of individuals from Barton-upon-Humber (PSN 641). Right: Example of the average preservation state of individuals from St James's Garden Burial Ground, London (PSN 510).

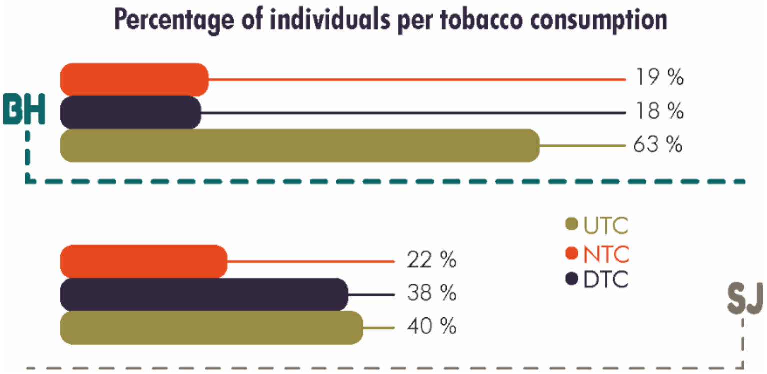

Fig. S3. Percentage of individuals in each tobacco consumption class after osteoarchaeological assessment. DTC: Detected tobacco consumer; NTC: Non-detected tobacco consumer; UTC: undetermined tobacco consumer.

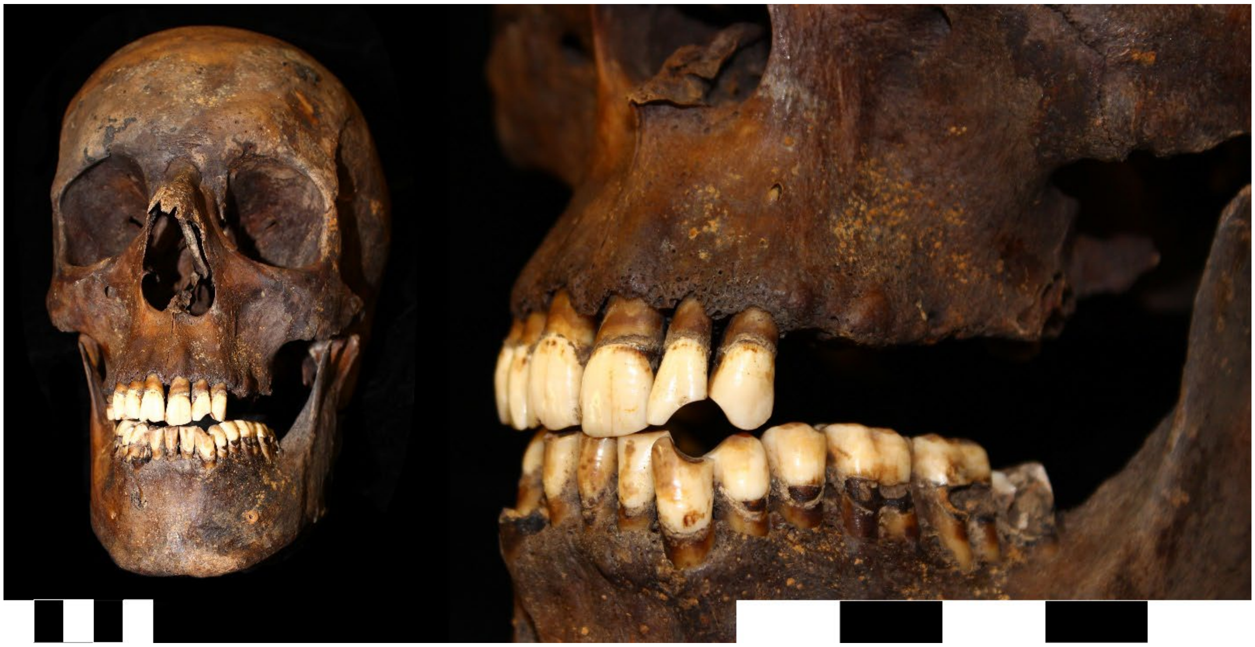

Fig. S4. Example of pipe notch evidence used for the classification of individuals into the tobacco consumption class group “Detected Tobacco Consumer”.

Evidence of a pipe notch in the dentition of a 35–49-year-old male from St James’s Garden Burial Ground (PSN 208). Left, picture of full skull. Right, detail of circular pipe notch between the second incisors and canine teeth.

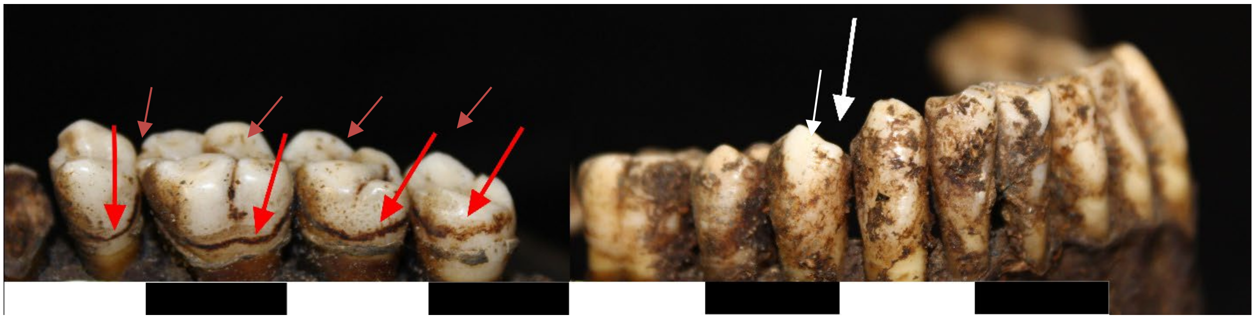

Fig. S5. Example of lingual staining and pipe notch evidence used for the classification of individuals into the tobacco consumption class group “Detected Tobacco Consumer”.

Evidence of lingual staining on the dentition associated with tobacco consumption in a 35–49-year-old male from St James’s Garden Burial Ground (PSN 191). Left, detail of lingual staining with red arrows indicating the staining. Right, detail of tooth wear from the use of a clay pipe, white arrow indicates the position of the pipe notch.

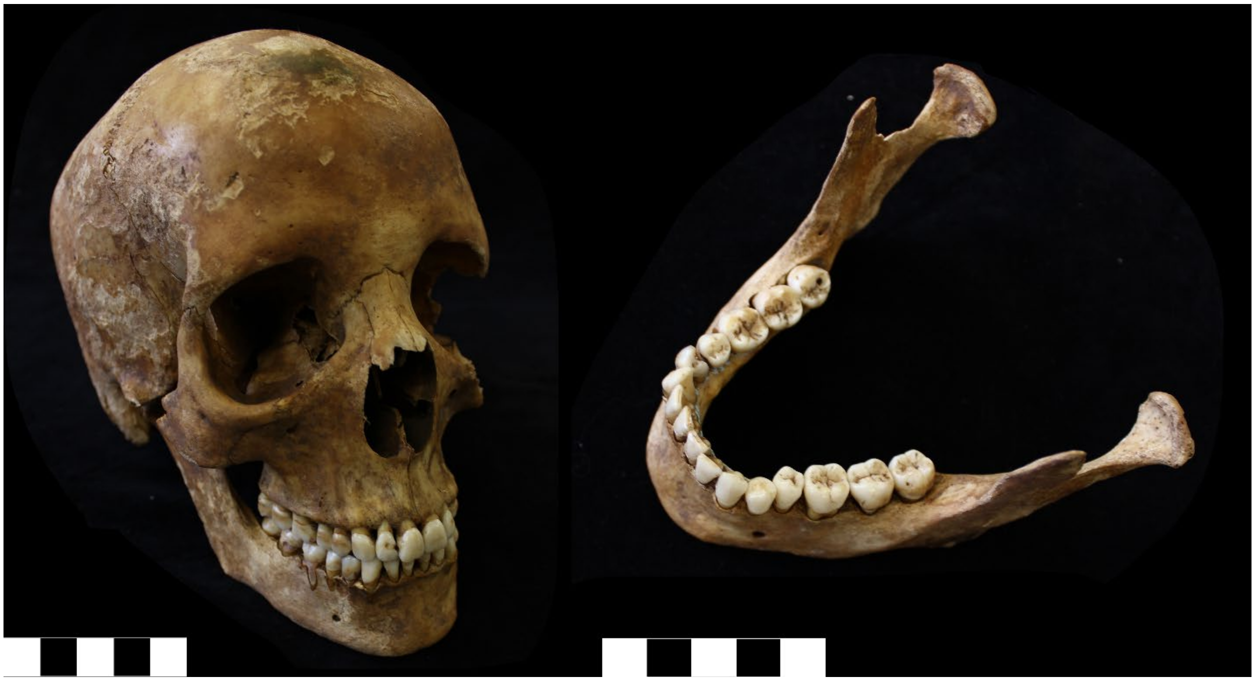

Fig. S6. Example of an individual with an absence of evidence for pipe notches and lingual staining, used for the classification of individuals into the tobacco consumption class group "Non-Detected Tobacco Consumer".

Example of an absence of evidence for lingual staining or tooth wear associated with tobacco consumption. Left, detail of full skull. Right, detail of the mandible and dentition with an absence of evidence for pipe-related tooth wear or lingual staining.

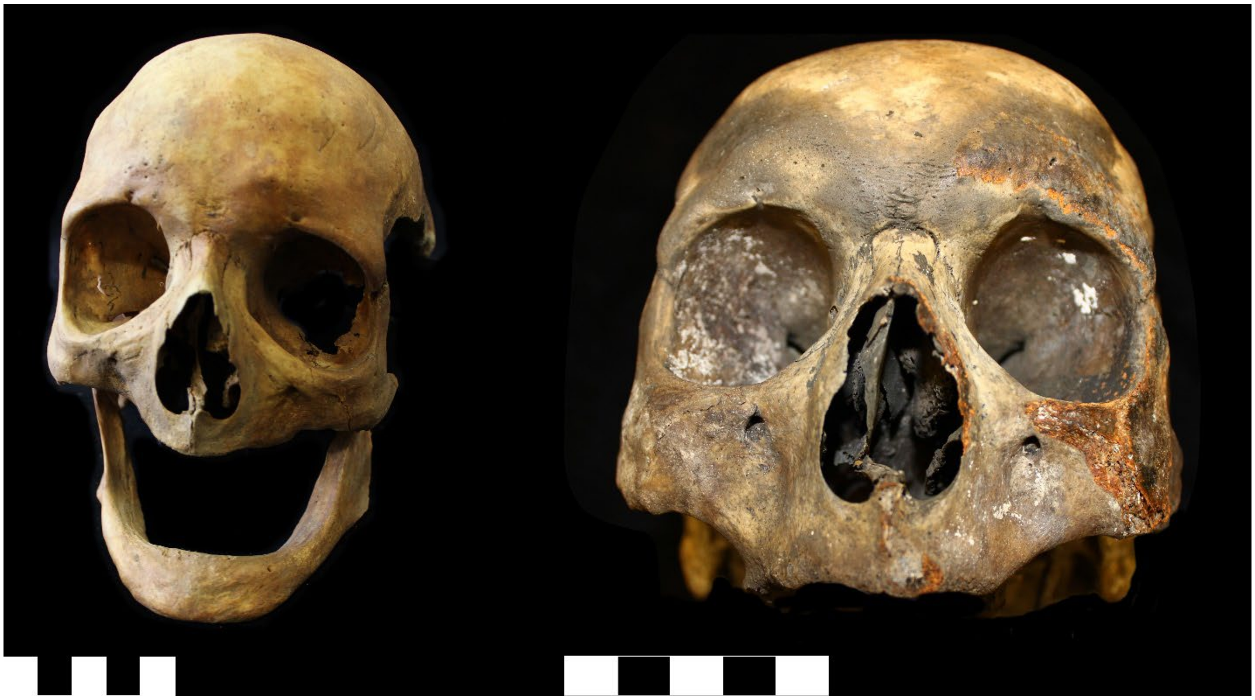

Fig. S7. Example of an individual unobservable for evidence of tobacco consumption due to antemortem tooth loss who was classified in the tobacco consumption class group "Undetermined Tobacco Consumption".

Examples of complete antemortem loss of dentition (edentulism). Left, detail of full skull of a 50+ year old possible female from Barton-upon-Humber (PSN 598). Right, upper jaw of a 35–49-year-old male from St James's Garden Burial Ground (PSN 280).

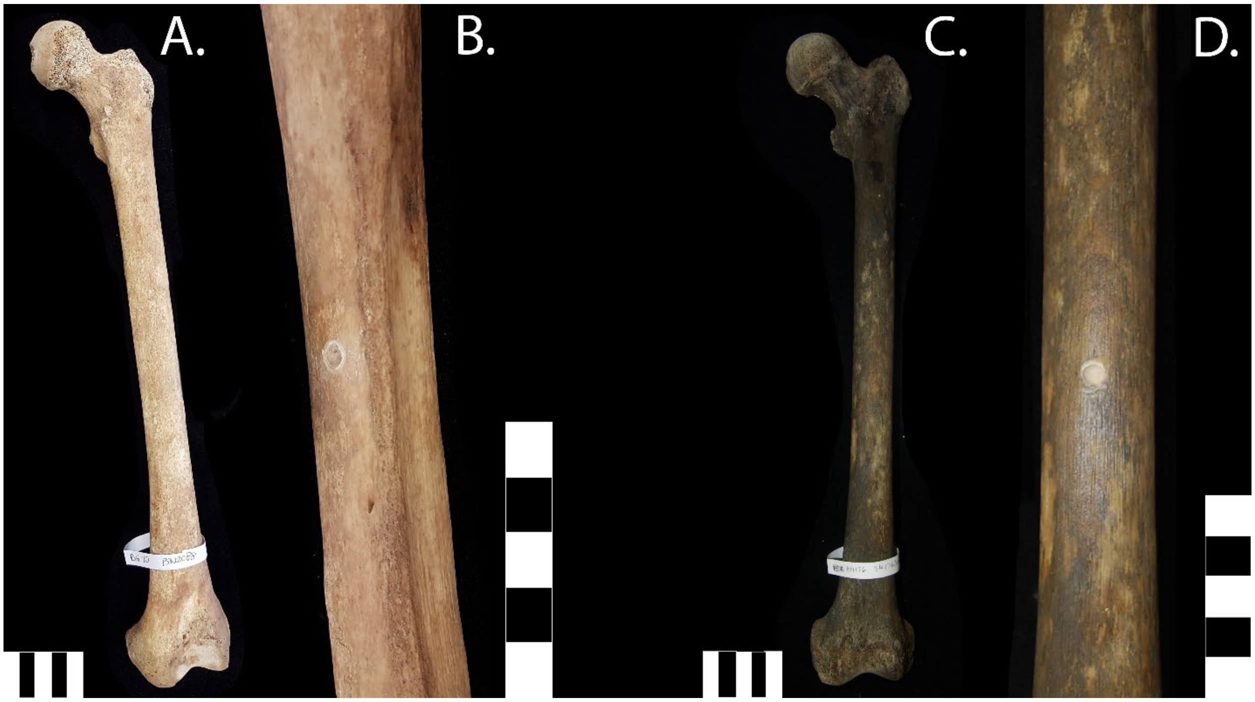

Fig. S8. Examples of femora before and after micro-sampling for the untargeted metabolomic study.

A&B) Example of the average condition of the bone from Barton-upon-Humber (PSN 616). C&D) Example of the average condition of the bone from St James's Garden Burial Ground (PSN 575). A&C) Full femur before sampling. B&D) Detail of femur mid-shaft after micro-sampling of cortical bone.

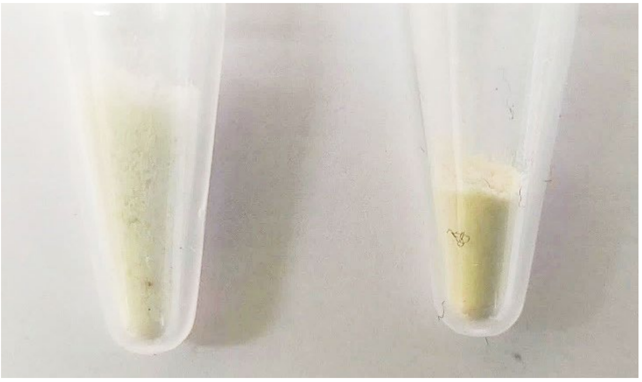

Fig. S9. Examples of resultant cortical bone micro-sample from the two archaeological skeletal collections. Left, Barton-upon-Humber. Right, St James's Garden Burial Ground.

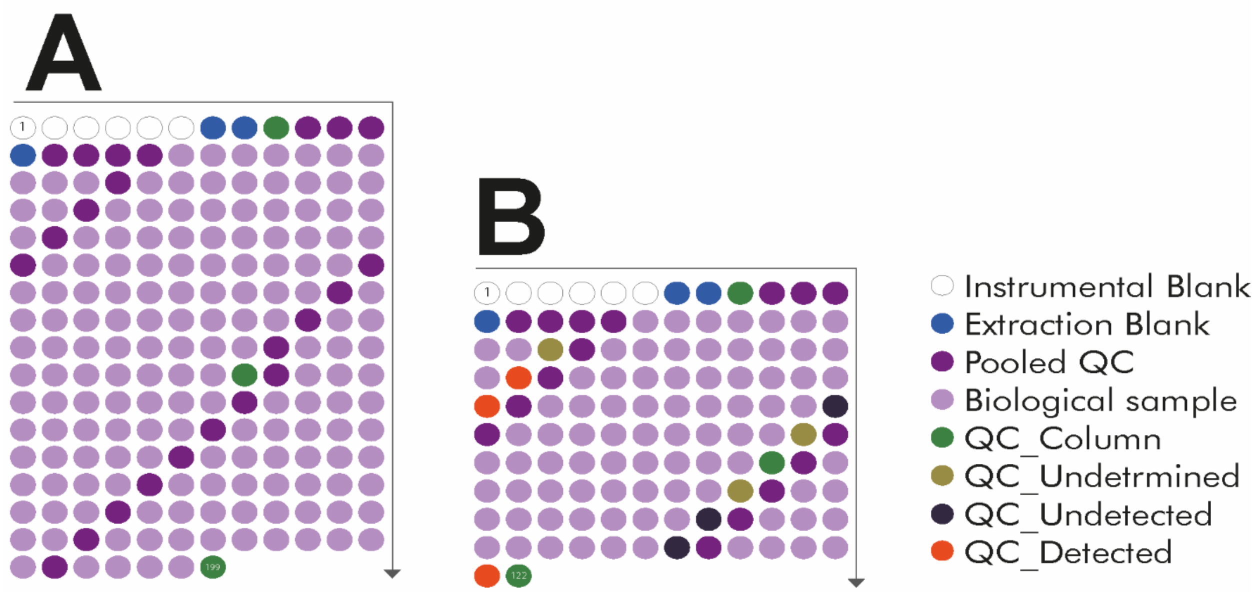

Fig. S10. Injection order for batch used on untargeted metabolomic LC-HRMS assays. A) Order for MS^E assay. B) Order for MS1 assay.

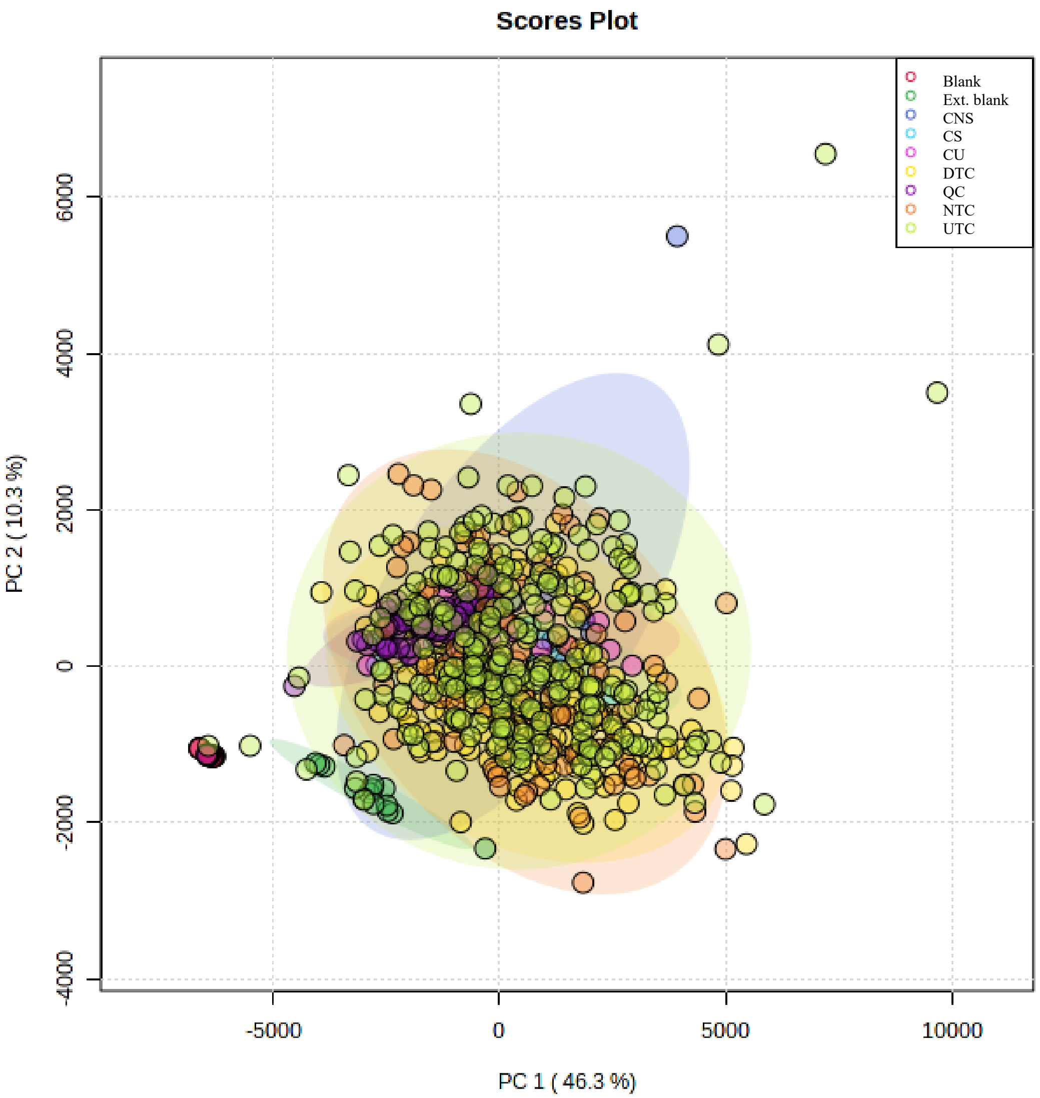

Fig. S11. PCA score plot for the RAW IM-TOF-MS data of the 6 batches of apolar/less polar metabolites separated using a C18 column, for the different samples (biological and QA) extracted from cortical bone in femora in British individuals.

Detected (DTC), individuals' samples with osteobiography record of smoking. Non detected (NTC), individuals' samples without osteobiography record of smoking. Undetermined (UTC), individuals' samples where it was not possible to record their osteobiography. CS, pooled sample from DTC samples. CN, pooled sample from NTC samples. CU, pooled sample from UTC samples. QC, pooled sample from Detected & Undetected samples. Blank, instrumental blanks. Ext. blank, extraction blanks.

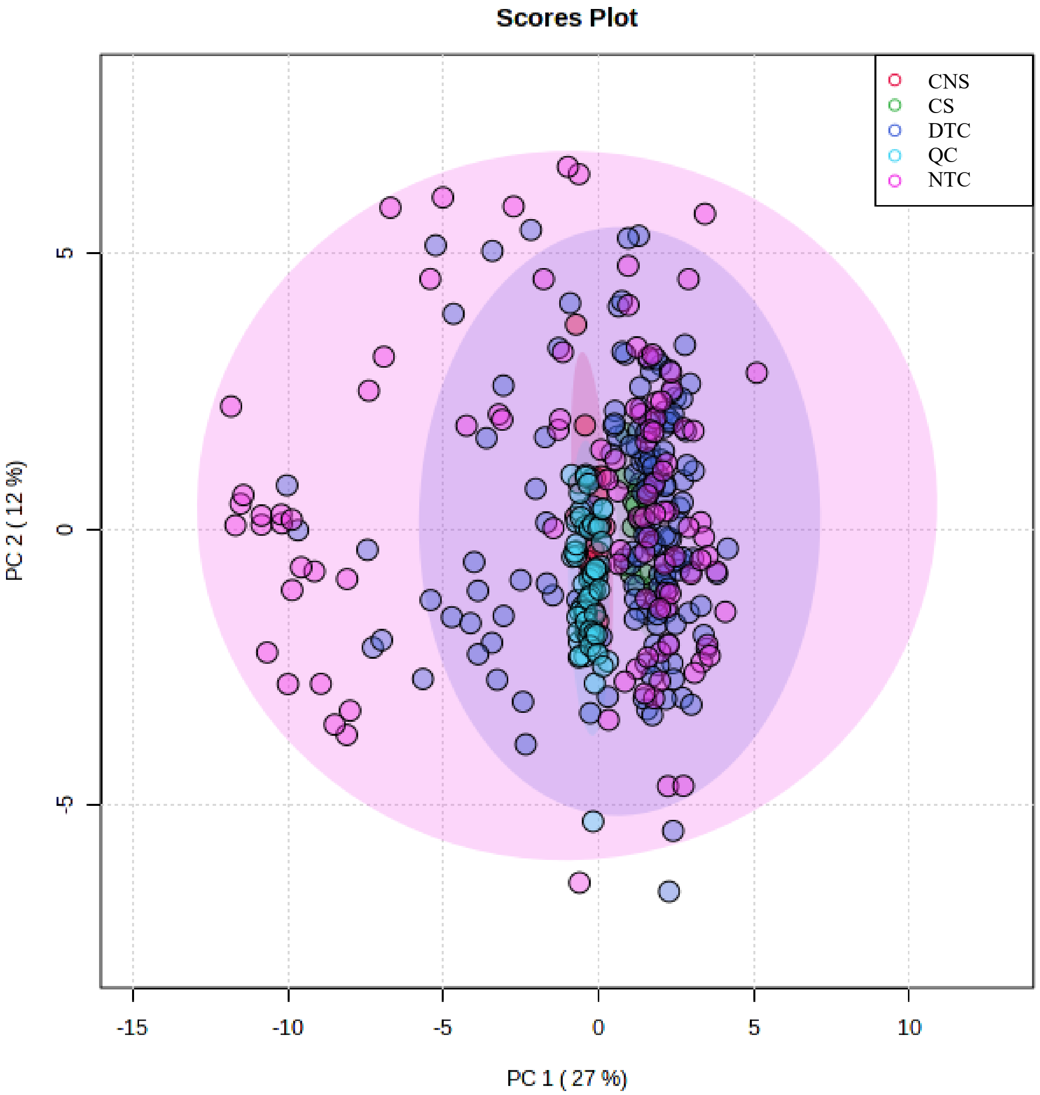

Fig. S12. PCA score plot for the IM-TOF-MS data of the 6 batches of apolar/less polar metabolites separated using a C18 column, for the different samples (biological and QC) extracted from cortical bone in femora in British individuals.

Statistical model made from MS data after data filtering and data normalization processes. Detected (DTC), individuals' samples with osteobiography record of smoking. Non detected (NTC), individuals' samples without osteobiography record of smoking. CN, pooled sample from detected class samples. CS, pooled sample from undetected class samples. QC, pooled sample from Detected & Undetected samples.

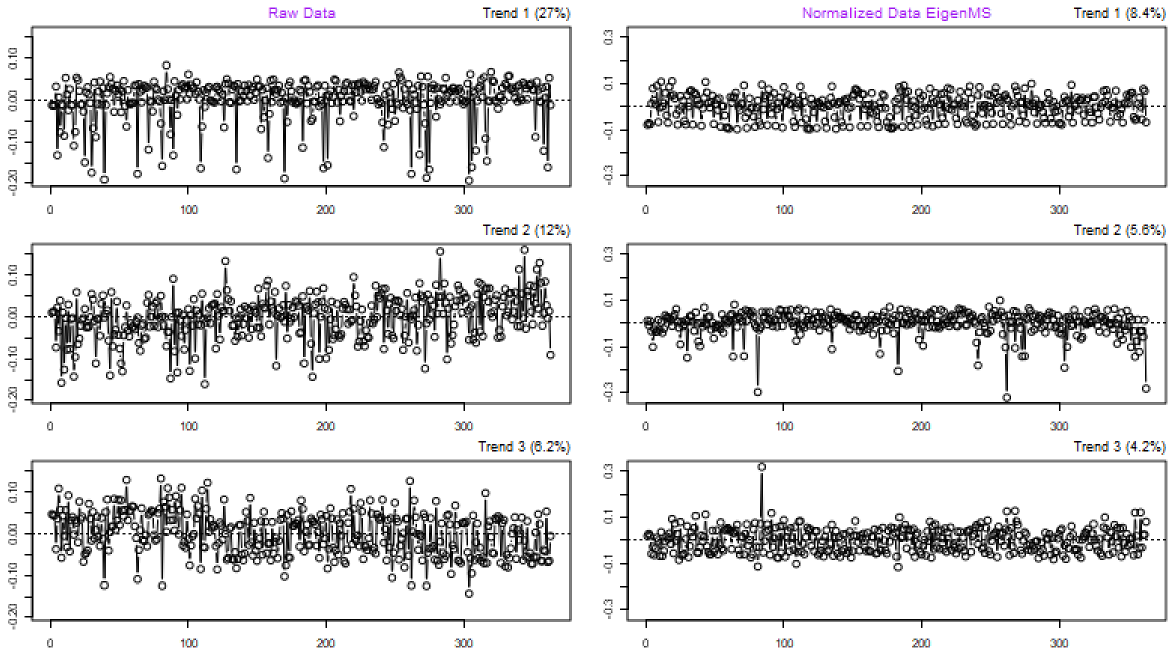

Fig. S13. Trend plot for all samples evaluated in the untargeted metabolomic study for cortical femora of British individuals.

Reduction of the variance on the main three trends before (left) and after (right) EigenMs batch effect correction algorithm was applied to the data.

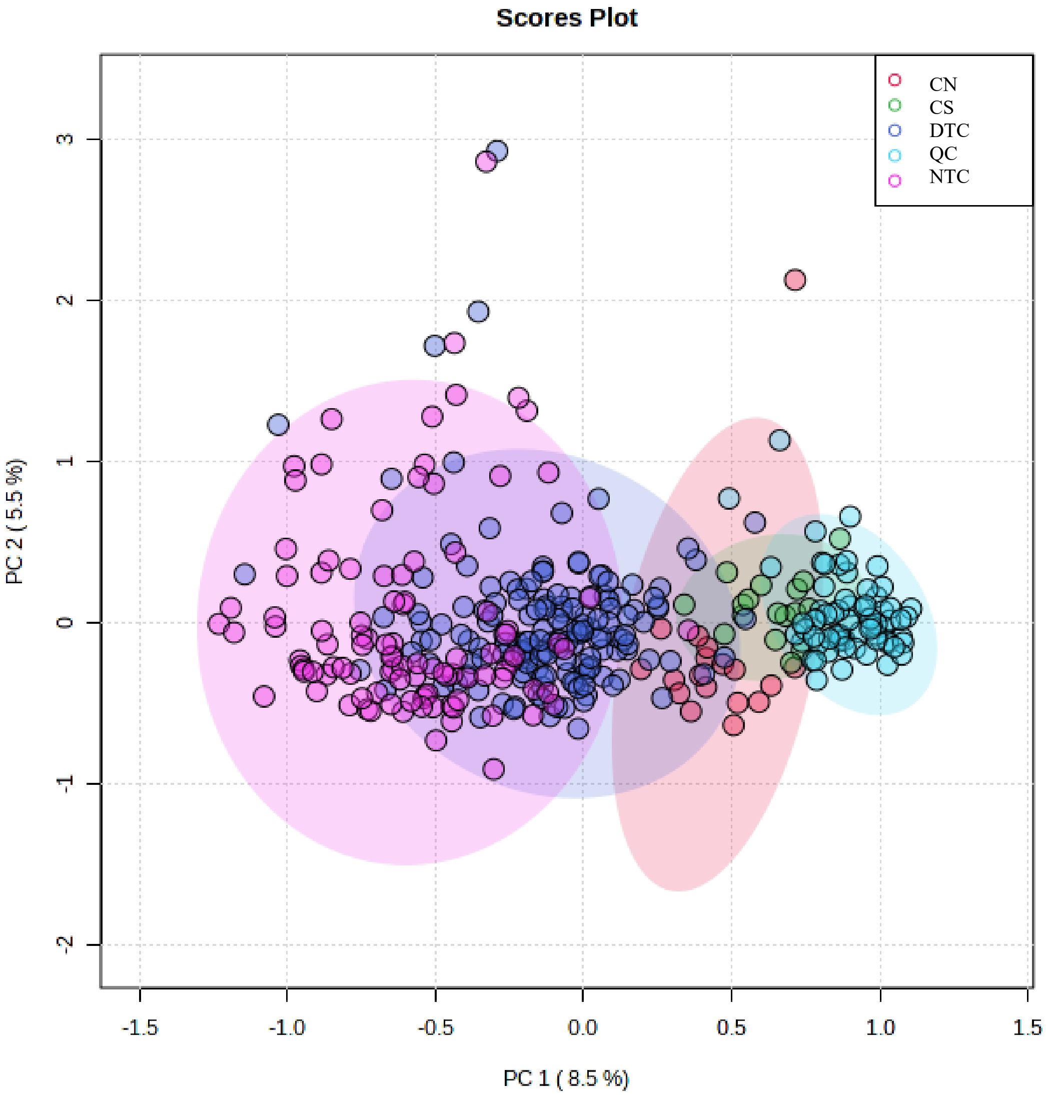

Fig. S14. PCA score plot for the filtered & normalized IM-TOF-MS data of the 6 batches after Eigen Ms batch correction of apolar/less polar metabolites separated using a C18 column, for the different samples (biological and QC) extracted from cortical bone in femora in British individuals.

Detected (DTC), individuals' samples with osteobiography record of smoking. Non detected (NTC), individuals' samples without osteobiography record of smoking. CN, Pooled sample from detected class samples. CS, Pooled sample from undetected class samples. QC, Pooled sample from NTC & DTC.

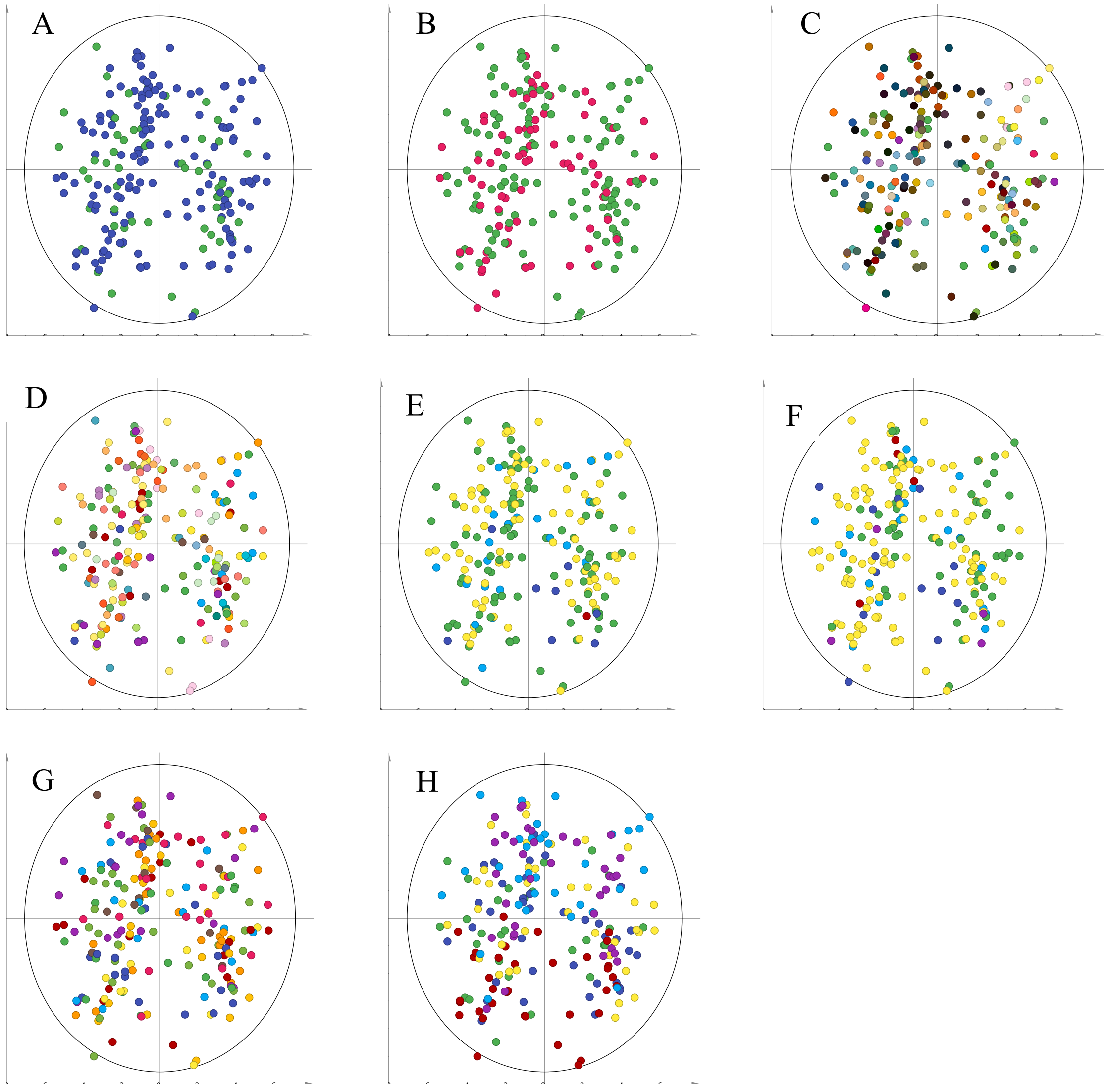

Fig. S15. PCA score plot for the filtered & normalized IM-TOF-MS data of the 6 batches after Eigen Ms batch correction of apolar/less polar metabolites separated using a C18 column, for the different samples (biological and QC) extracted from cortical bone in femora in British individuals.

A) samples coloured by site, green Barton-upon-Humber, blue, St. James B) samples coloured by leg side, green left, red, right C) samples coloured by weight D) samples coloured by circumference E) samples coloured by age F) samples coloured by biological sex G) samples coloured by extraction batch H) samples coloured by injection batch.

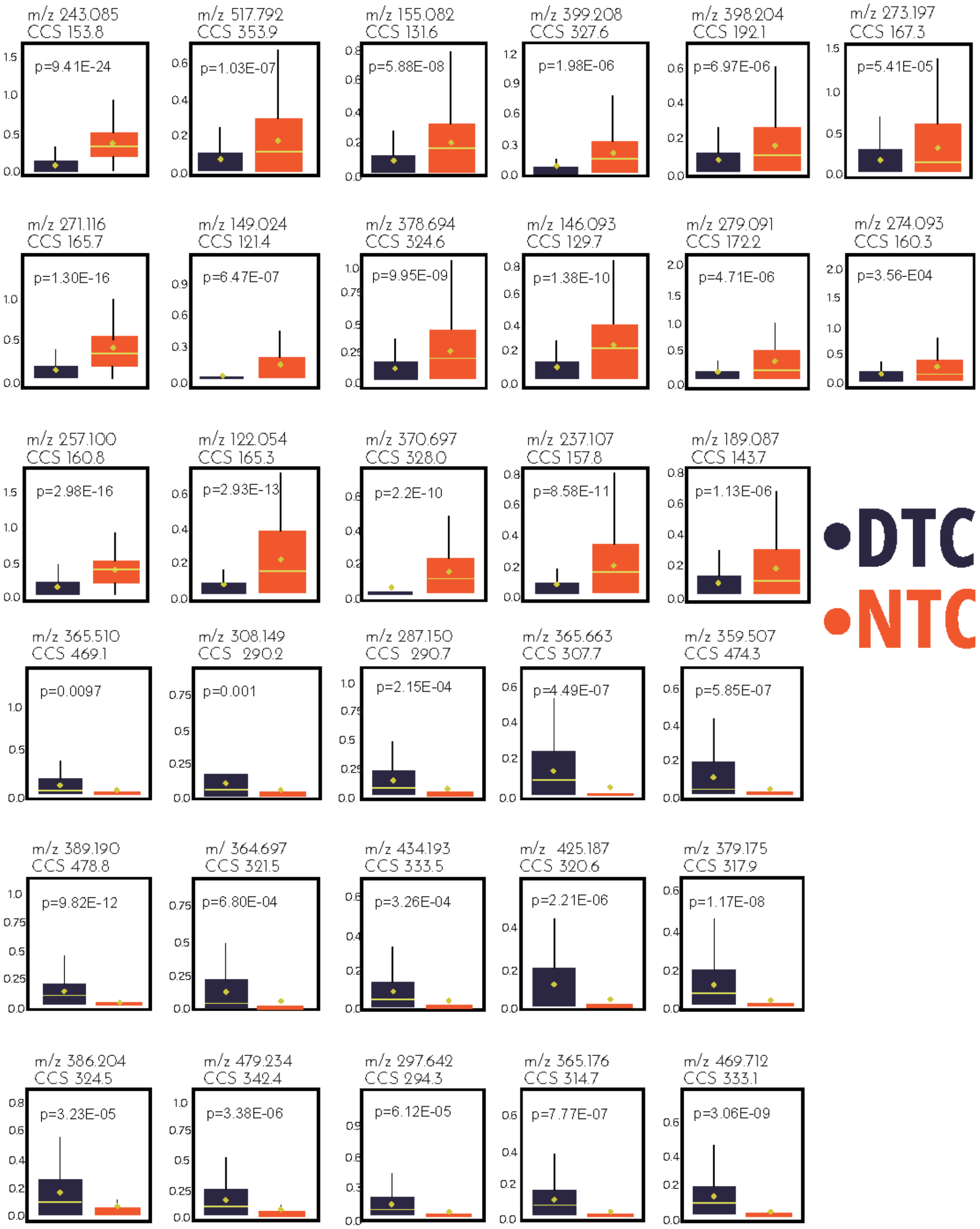

Fig. S16. Box plots for significantly up regulated features on the untargeted metabolomics of cortical bone of British human skeletal remains according to Volcano plot. m/z: mass to charge ratio. CCS: collision cross section (Å^2).

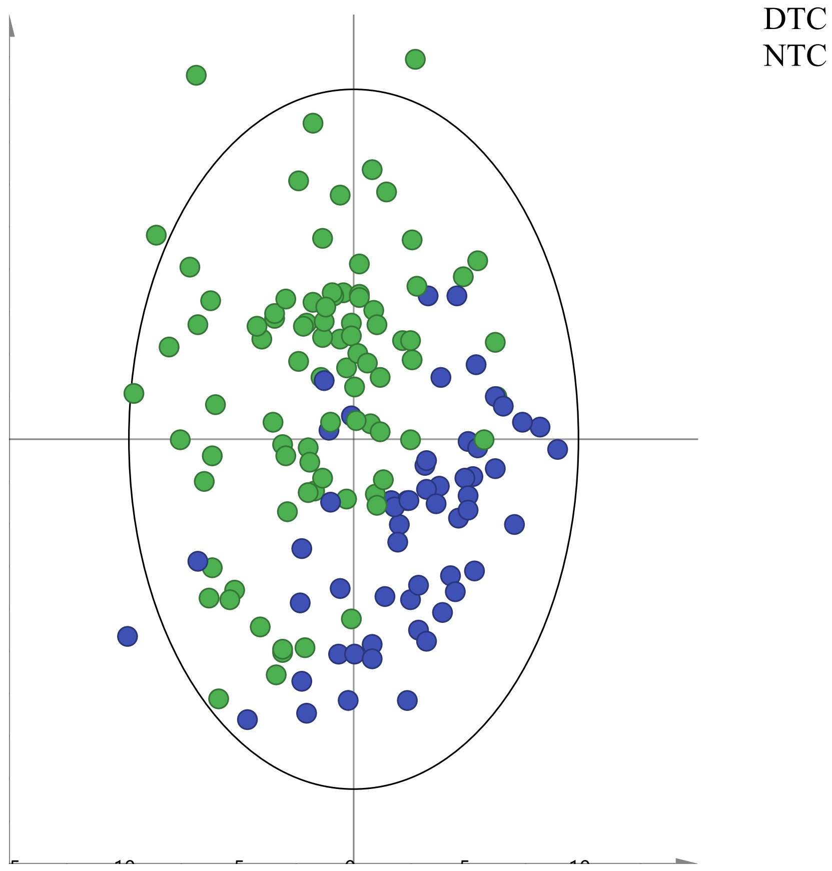

Fig. S17. PCA score plot for the IM-TOF-MS data of the MSe batch of apolar/less polar metabolites separated by C18 column, for the different samples (biological and QC) extracted from cortical bone in femora in British individuals.

Statistical model made from MSe data after data filtering and data normalization processes. n=142, variables=376. PC1: R2X:0.0413 Q2:0.0141 PC2: R2X:0.0293 Q2:0.000968

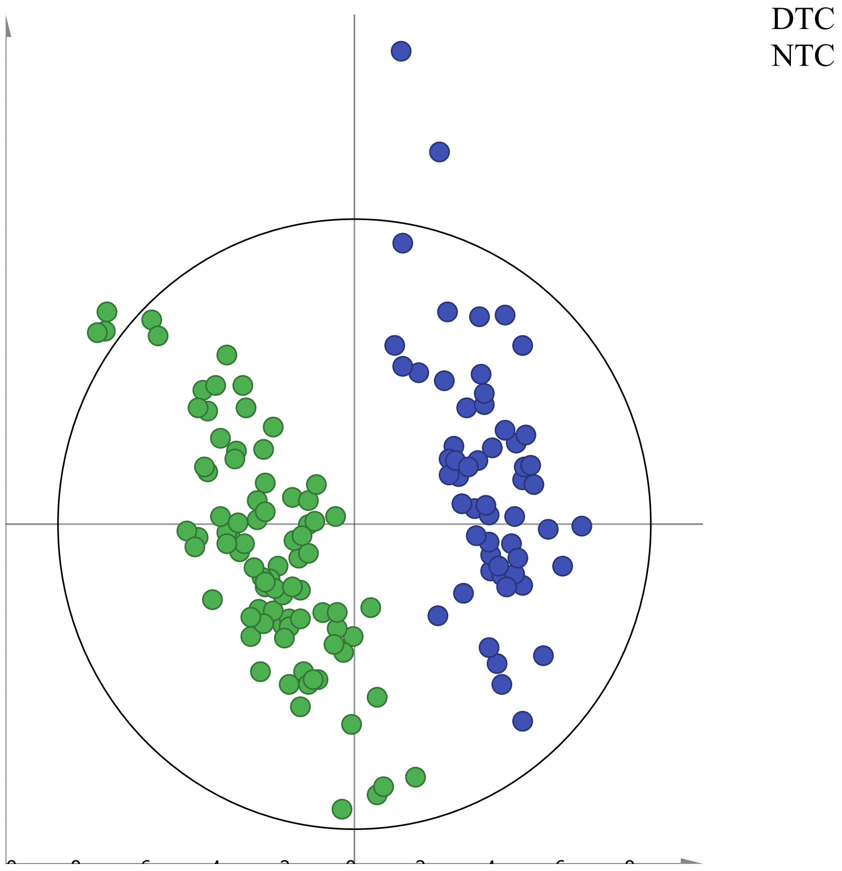

Fig. S18. PLS-DA score plot for the IM-TOF-MS data of the MSe batch of apolar/less polar metabolites separated by C18 column, for the different samples (biological and QC) extracted from cortical bone in femora in British individuals.

Statistical model made from MSe data after data filtering and data normalization processes. n=142, variables=376. PC1: R2X:0.034 R2Y:0.8 Q2:0.667 PC2: R2X:0.0289 R2Y:0.103 Q2:0.124.

m/z	Neutral mass (Da)	Charge	Retention time (min)	CCS (Å^2)
**229.11942**		1	0.714	152.3272
**286.14242**	222.12665	1	0.714	165.2758
**146.09371***		1	0.895	129.6561
**286.14182***		1	0.920	166.9868
**414.19998**		1	1.799	189.0990
**155.08237***		1	1.954	131.5992
**312.50725**		3	2.185	420.3927
**287.14986****		2	2.549	290.6829
**609.25631**		1	2.780	227.2362
**364.69787****		2	2.805	321.4717
**398.20382***		1	2.884	192.1175
**136.07629**		1	2.960	130.4217
**409.21958**		1	2.960	201.2754
**421.71844**	797.45843	2	3.013	333.6902
**365.51061****	1049.54610	3	3.090	469.0856
**389.19034****	1120.58530	3	3.090	478.8497
**189.08706***		1	3.115	143.6907
**386.20015****		2	3.115	327.7460
**359.50754****	1031.53691	3	3.168	474.2830
**399.20835***	385.19679	2	3.245	327.5591
**433.73446**		2	3.270	327.1170
**297.64167****		2	3.323	294.2707
**365.17570**		2	3.323	314.6704
**379.17457****		2	3.323	317.8737
**469.71217****		2	3.323	336.2909
**320.17239**		1	3.349	174.3878
**434.19314****		2	3.374	333.5361
**421.71925**		2	3.374	333.6902
**479.23443****	912.49042	2	3.425	342.3783
**370.69653***		2	3.580	327.9858
**425.18826****		2	3.633	323.9441
**378.69444**		2	3.735	324.5776
**308.14903****		2	3.890	290.2220
**365.66344****	729.31233	2	3.890	307.6732
**378.69430***		2	4.020	324.5776
**547.26459**		2	4.098	365.1339
**517.79206***		2	4.279	353.9552
**237.10676***	236.09948	1	5.440	157.7871
**149.02513***		1	7.508	121.3549
**243.08498***		1	7.714	155.7017

**257.10096***		1	7.869	160.8029
**279.09719***		1	7.894	172.1815
**271.11651***		1	7.998	165.6836
**285.13183**	246.16867	1	8.153	170.3690
**446.19820**		2	8.204	336.5473

Table S1. List of molecules with a VIP >1 from the PLS-DA model for less polar/apolar metabolites assay through LC-HRMS.

*Up regulated DTC. **Up regulated NTC

Phase	Total samples	% DTC	% NTC
**Barton-upon-Humber**			
**BH1**	33	9.1	90.9
**BH2**	85	65.9	34.1
**St James cemetery, London**			
**SJ1**	12	50	50
**SJ2**	24	58.3	41.7
**SJ3**	16	68.8	31.3
**SJ4**	17	17.6	82.4
**SJ N.A.**	43	67.4	32.6

Table S2. List of archaeological phases studied and respective percentage of individuals from undetermined group classified as DTC or NTC after using the ROC-SVM model with the molecules with a VIP >1 from the PLS-DA model for less polar/apolar metabolites assay through LC-HRMS.

Data S1. (separate file)
Metadata for the different samples studied and MS1/MSe injection lists (.xls).

Data S2. (separate file)
Untargeted metabolomic data matrix for the features with VIP>1 on PLS-DA model for QA and biological samples (.xls).

Data S3. (separate file)
Annotation candidates for the features with level 3 identification with VIP>1 on PLS-DA model on the untargeted metabolomic assay (.xls).

Data S4. (separate file)
Assignation list for the different "UTC" class individuals after using the ROC-SVM model for their classification (.xls).
